# Comparison of pharmaceutical pricing and reimbursement systems in Turkey and certain EU countries

**DOI:** 10.1186/s40064-016-3455-z

**Published:** 2016-10-26

**Authors:** Enver Kagan Atikeler, Gulbin Özçelikay

**Affiliations:** 1 Faculty of Pharmacy, Hacettepe University, Ankara, Turkey; 2 Faculty of Pharmacy, Ankara University, Ankara, Turkey

**Keywords:** European Union pharmaceutical pricing systems, Health policy, Pharmaceutical pricing, Reference pricing, Reimbursement

## Abstract

Recently, the need for health care services has increased gradually and the limitations in sources allocated for this area have been recognized. Moving from this fact, it has gained a supreme importance to determine what health programs or technologies will be given priority. According to Danzon (Reference pricing: theory and evidence, reference pricing and pharmaceutical policy: perspectives on economics and innovation, springer, New York, pp 86–126, 2001), arrangements towards controlling the expenses through price and profit controls, reimbursement methods and incentives have recently gained wide currency. This present study examines; along with the current situation in Turkey, pharmaceutical pricing methods, reimbursement methods and basic health indicators, within the scope of changing pharmaceutical policies, in Turkey, the EU countries which Turkey takes as reference and the United Kingdom, the implementations of which are of utmost importance for other countries. Upon the research conducted, it was detected that the pharmaceutical pricing in Turkey has been performed on the basis of the reference pricing system that takes Italy, Portugal, Spain, Greece and France as reference. The regulations regarding the reimbursement process are determined by SSI. For Turkey’s case; pricing and reimbursement system has been changed numerous times and the discount rates has incrementally risen. In pricing, on the other hand, during this period companies faced with difficulties in economic terms because of the fact that price discount of high rates are implemented over the reference price and that the European currency of Euro is determined as 70% of previous year average Euro sales rate which is 2,1166 for the year 2016. Each country has specific regulations and pricing and reimbursement policies of medicines based on economic situation, reimbursement methods and market size. The aim of pricing and reimbursement systems are reaching more efficient and sustainable healthcare systems.

## Background

As an important part of health service delivery, pharmaceutics is an industrial branch that produces simple or compound pharmaceutical forms within the framewoks of pharmaceutical technology and in compliance with the scientific standards from chemicals of synthetic, vegetal, animal and biological origin to be used for therapeutic, preventive and nutritive purposes in industrial, human and veterinary medicine (DPT 2001).

**Fig. 1:**
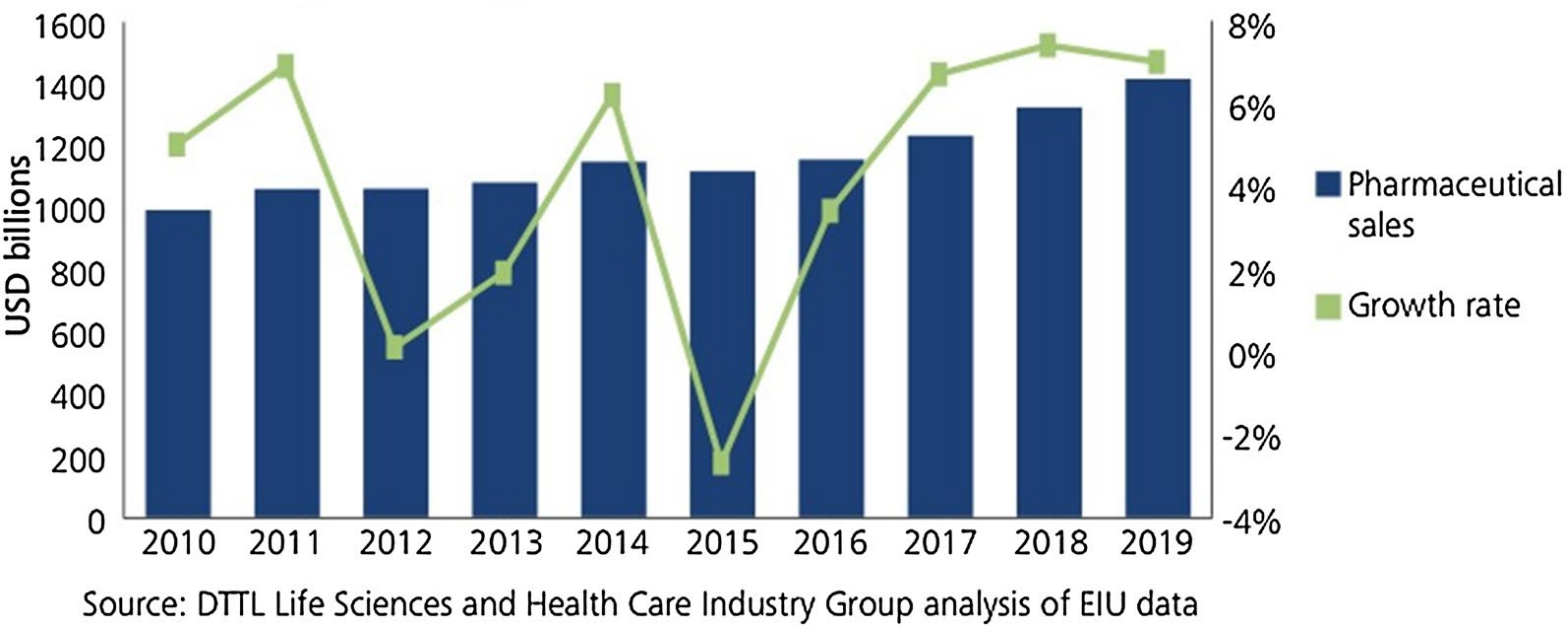
Global pharma segment sales *Source*: DTTL Life Sciences and Health Care Industry Group analysis of EIU data

In its World Drug Report published in 2011, WHO states that the disease load of countries has shifted from acute diseases to chronic diseases. For this reason, difficulties are experienced in supply of medicines, and medicine utilization is also affected. While the size of pharmaceutical industry was 731 billion dollars in 2007, this Fig. 1 increased to 965 billion dollars in 2011 (SCRIP100 2014), over 1.000 billion for 2015 and expected to be 1.400 billion dollars by 2019 (Deloitte 2016).

Recently, the need for health services has increased gradually and the limitations in sources allocated for this area have been recognized. Moving from this fact, it has gained a supreme importance to determine what health programs or technologies will be given priority. According to Danzon (2001), arrangements towards controlling the expenses through price and profit controls, reimbursement methods and incentives have recently gained wide currency. Despite those were the main reasons of health refoms, there are also other reasons that are related to the improvements in social and health areas to lead to the generation of new reforms. Governments started to search actions to decrease health and education expenditures because of financial crises that occurred by end of 1970s. With the separation of service providers and service funding in the health system, and the development of policies aimed at restraining public expenditure, it has been planned to minimize presence of public sector in both structures (Çaliskan 2009). The most commonly used method to bring pharmaceutical expenditures under control is the reference pricing system, which is used in numerous countries. The reference pricing system is the determination of the ceiling price of reimbursement for a certain group of medicines by the payer organization or insurance company. It is assumed that such a practice concerning medicines will lead to a decrease in medicine expenditures as medicine prices decrease (Çaliskan 2008).

While innovation in the pharmaceutical industry is usually welcomed by all stakeholders, it inevitably comes at the expense of having higher medicine prices and growing health-related expenditures (Rapple et al. 2010).

**Fig. 2:**
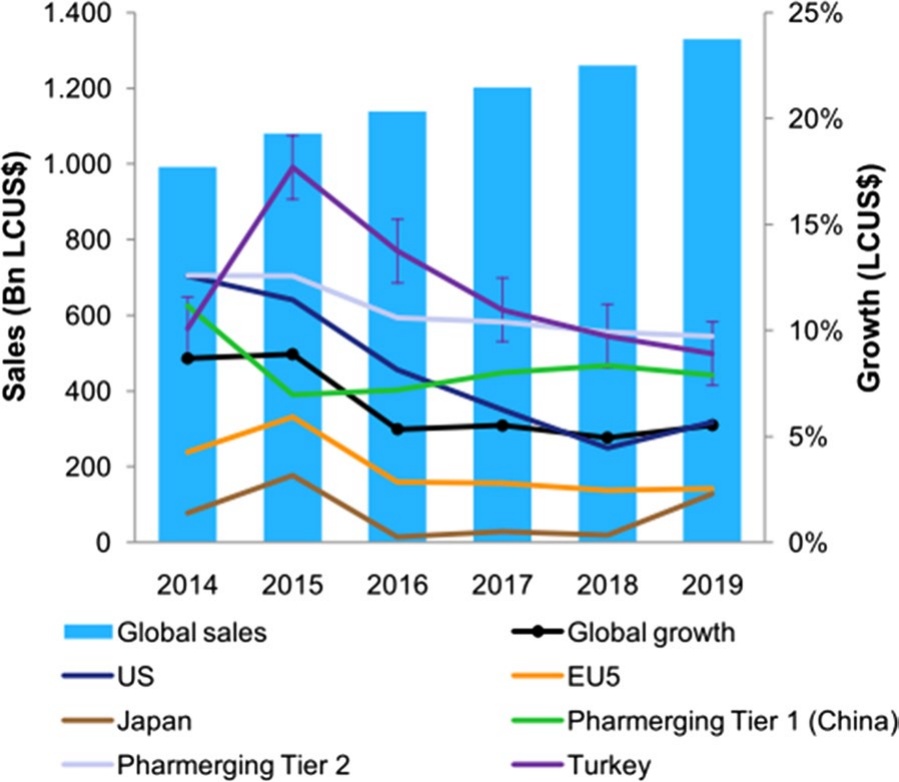
Expected growth rate *Source*: IMS Market Prognosis Sept. 2015; at ex-manufacturer price level, without rebates and discounts

Global growth expected to be 5–8% by 2019 and reaches 1.300 billion US dollars, growth expected to be 11–14% in Turkey (IMS 2015) (Fig. 2).

**Table:** 

Developed markets CAGR 2015–2019 (%)		Pharmerging markets CAGR 2015–2019 (%)	
US	6 to 9	Tier 1 (China)	6 to 9
Japan	0 to 3	Tier 2	9 to 12
Germany	2 to 5	Brasil	10 to 13
UK	4 to 7	India	11 to 14
France	−2 to 1	Russia	6 to 9
Italy	3 to 6	Tier 3	4 to 7
Canada	3 to 6	Turkey	11 to 14
Spain	2 to 5		
Developed	4 to 7	Pharmerging	6 to 9

*Source*: IMS Market Prognosis Sept. 2015; at ex-manufacturer price level, without rebates and discounts

Pricing of pharmaceuticals and reimbursement has always important argument for countries. Pricing and reimbursement systems are closely linked to the realisation of European policy objectives such as the internal market, pharmaceutical competitiveness, sustainable research and development, and the protection of human health. Each country uses different schemes and policies, adapted to its own economic and health needs. These national systems are regularly reviewed or adapted in order to take account of political priorities, market evolutions, and patients’ needs. The variety of healthcare and social security systems in the EU has an impact on the pharmaceutical industry, wholesalers, pharmacists, doctors, health insurers, and patients.Our article aims to show pricing and reimbursement systems in different countries. (European Union 2016).

One of the main driven pricing methods for pharmaceuticals are external reference pricing and internal reference pricing. External reference pricing (International price comparison); the practice of using the price(s) of a medicinal product in one or several countries in order to derive a benchmark or reference price for the purposes of setting or negotiating the price of the product in a given country.

Internal reference pricing; commonly employed in EU countries as a means to regulate out-of-patent drug prices. Describes the practice of setting the price to be paid by public payers by comparing prices of equivalent or similar products in a chemical, pharmacological or therapeutic group.c The ‘reference price’ applies to all pharmaceuticals within the corresponding group of products (Ruggeri and Nolte 2013).

## Methods

This study was conducted with a descriptive method. The main tool of the study is implemented regulations of pharmaceutical pricing and reimbursement in Turkey, Italy, France, Portugal, Spain, Greece and United Kingdom. 5 Countries (Italy, France, Portugal, Spain, Greece) selected based on reference countries on pricing of Turkey and UK selected with reason of one of the leading country on health policy. Data sources consist of Guidelines of Health Authorities and web pages, related articles, reports, other guidelines and laws and directives. Data are limited to 2015 April for countries mentioned in the article and June 2016 for Turkey.

## Results

As shown in Table 2, there are differences in reimbursement rates of comparison countries. While in France, Spain and Italy basically the reimbursement rates are set according to medical and economic evaluations, in UK free pricing is applied. By conducting margin control, however, a price intervention is carried out with direct control for the drugs that exceed the expected cost determined by PPRS agreements or that do not provide the expected benefit with their therapeutic effectiveness. While Portugal set reimbursement rates according to disease groups, Turkey and Greece apply the same control method for all drugs and pay lower than the defined lowest reference price in reimbursement system. Price comparison with other countries such as internal or/and external reference pricing is a criterion for all countries but in Turkey and Greece, it is the main criterion and the ceiling price is determined on the basis of the lowest price in other countries. A lower price may be defined upon the request of manufacturer.

Pricing is basically processed by using 3 different methods

- Free Pricing (Germany, The Netherlands, Denmark)
- Direct Price Controls (France, Italy, Portugal, Greece, Spain, Turkey, Belgium)
- Margin Controls (UK)

As shown in Tables 1 and 2 medicines has free pricing in UK but the prices are controlled through margin controls. On the other hand, all other countries examined in our study apply direct price controls. France and Italy define prices upon negotiations with companies. Prices are not determined only with upon effectiveness and comparisons with other countries. In price determination, there are other factors considered such as production costs, therapeutic efficiency, R&D expenditures, sale amounts, advertising costs and contribution to the economy of the country (Balçik and Karsavuran 2012).

**Table 1:** Comparison of pricing methods in Turkey and certain other countries (Mossialos et al. 2004)

Country	Market	Direct price controls	Use of international price comparisons	Margin controls	Reference pricing
France	Original	Yes	Yes	No	No
	Generic	No	Yes	No	Yes
Greece	Original	Yes	Yes	No	Yes
	Generic	Yes	Yes	No	Yes
Italy	Original	Yes	Yes	No	No
	Generic	No	Yes	No	Yes
Portugal	Original	Yes	Yes	No	No
	Generic	No	Yes	No	Yes
Spain	Original	Yes	Yes	No	No
	Generic	No	Yes	No	Yes
United Kingdom	Original	No	No	Yes	Available for drugs excluded from PPRS
	Generic	Yes	No	No	Available for drugs excluded from PPRS
Turkey	Original	Yes	Yes	No	Yes
	Generic	Yes	Yes	No	Yes

**Table 2:** Reimbursement systems in Turkey and certain other countries

Country	Reimbursement levels	Generic—original difference	Reimbursement of prescription and non-prescription drugs	Negative and positive list
France	Major, moderate, weak	N/A	No reimbursement for non-prescription drugs	Available
Greece	0, 10 and 25%	Available	No reimbursement for non-prescription drugs	N/A
Spain	100, 90, 60 and 0%	N/A	Some non-prescription drugs are reimbursed	Available
Italy	Class A and H 100%, class C and C-bis 0%	N/A	No reimbursement for non-prescription drugs	N/A
Portugal	A 95%, B 69%, C 37%, D 15%	N/A	No reimbursement for non-prescription drugs	Available
United Kingdom	PPRS price negotiation	Available	No reimbursement for non-prescription drugs	Available
Turkey	Generally 41 and 28% discount. 18 and 10% available for 20 year old medicines and generics generally	Available	No reimbursement for non-prescription drugs	Available

In Italy, Average Europe Price is taken into consideration while the prices are determined, but other factors mentioned above has impact on pricing decisions as well. Price comparisons are important for all the reviewed countries except UK, but this condition is not the sole criterion for other countries reviewed in our study except Turkey and Greece. Contribution margin and private health insurances exist in all countries. Turkey and Greece takes minimum price as reference whereas Portugal takes the average of the lowest 3 prices while defining prices. Price discounts are applied at variable rates in all countries. These discounts may continue to be applied in accordance with the changes occurring in yearly budgets (Tables 3, 4).

**Table 3:** Turkey, reference countries and UK pricing and reimbursement methods Source: Bentes et al. (2004), Caldeira et al. (2010), Donatini et al. (2001), Ganse et al. (2008), Gülergün et al. (2013), Kullman (2010), Lopes et al. (2011)

Country	Turkey		France		Greece		Spain		Italy		Portugal		UK	
Pricing policy	Statutory pricing		Price negotiation		Statutory pricing		Statutory pricing		Price negotiation		Statutory pricing		Margine control	
Direct price controls	Org	Gen	Org	Gen	Org	Gen	Org	Gen	Org	Gen	Org	Gen	Org	Gen
	Yes	Yes	Yes	No	Yes	Yes	Yes	No	Yes	No	Yes	No	No	Yes
Reference pricing	Yes		Yes		Yes		Yes		Yes		Yes		Only for PPRS-excluded medicines	
External reference pricing	Yes		Yes		Yes		Yes		Yes		Yes		No	
Internal reference pricing	Yes		Yes		Yes		Yes		Yes		Yes		Yes	
Different pricing for generics	Yes		Yes		Yes		Yes		Yes		Yes		Yes	
Price control	All medicines		Only for reimbursed medicines		All medicines		Only for reimbursed medicines		Only for reimbursed medicines		All prescription medicines		Only for medicines in NHS list	
Level of price control	Manufacturer price		Manufacturer price		Manufacturer price		Manufacturer price		Manufacturer price		Manufacturer price		NHS list price	
Reimbursement catagories	In general 41% and 28% discount. 18% and 10% available for 20 year old medicines and generics generally		Major (65%), moderate (35%), weak (0%)		0, 10 and 25%		100, 90, 60 and 0%		Class A and class H 100%, Clas C and class C-bis 0%		Class A 95%, B 69%, C 37%, D 15%		After PPRS price negotiation 100%	
Differences for originals and generics on reimbursement level	Yes		No		No		Yes		No		No		Yes	
Negative positive list	Yes		Yes		No		Yes		No		Yes		Yes	
Decision taking committees	Reimbursement commission of social security institution (SSI)		Committee of transparancy and economics		Transparancy committee		Inter-ministerial pricing committee		Technical committee under italian medicines agency (AIFA)		Portugal medicines agency		Health department of PPRS

**Table 4:** Reimbursement rates in Turkey Source: IMS Market Prognosis Sept. 2015; at ex-manufacturer price level, without rebates and discounts. Pharmaceutical Manufacturers Association of Turkey (IEIS 2016)

Discount rates (TL)	Original products		Generic products (%)	Twenty year old product		
Ex factory price ≤ 3.83	0%		0	0%		
3.84 ≤ Ex factory price ≤ 7.32	Without generic	With generic	10	0%		
	10%	10%				
7.33 ≤ Ex factory price ≤ 11.02	11% (New molecules for first year) 31%	18%	18	Local	With reference price	Without reference price
				10%	10%	10%
11.03 ≤ Ex factory price	11% (New molecules for first year) 41%	28%	28	28%	28%	40%

IMS Market Prognosis Sept. 2015; at ex-manufacturer price level, without rebates and discounts

Social Security Institution (SSI) is payer and decision taking body for drug reimbursement. Discount rates applies on ex-factory prices as shown below and determine the reimbursement price.

## Discussion

Recently pharma industry is a global industry that has the highest R&D potential. On one hand discovery of new molecules for specific and malignant diseases, and the transformation of extended life expectancy into a better quality of life on the other, cross-border competition of global economy, increasing state intervention and control, leads to differentiation of pharma industry from other industries (TOBB 2008).

The starting point of drug policy determination is defining the target. This varies according to the income level each country. On the other hand, while defining this policies it should be noted that pharmaceutical innovation saved millions of life and prolonged life expectancy during the past century (TİTCK 2014).

When pricing and reimbursement system is reviewed for Turkey and other countries in our study; it can be seen that each country has its own special conditions. While Turkey has some similarities with France, Greece, Italy and Spain with respect to pricing and reimbursement, it has significant differences from UK pricing and reimbursement system. In UK, pricing and reimbursement process go hand in hand and there is a margin control-based system existing.

Margin control-based system used in the UK; describes a profit framework which is negotiated periodically between the Department of Health and the pharmaceutical industry (Pharmaceutical Price Regulation Scheme, PPRS) (Ruggeri and Nolte 2013).

Since the PPRS is not a guaranteed profit scheme, companies should not be penalised if they introduce a new, clinically and cost effective medicine which finds high acceptance by patients and prescribers. Equally, companies are not guaranteed a continuing profit level if sales decline under normal commercial circumstances. In recognition of this, the PPRS provides an element of flexibility in assessing company profitability. This is called the Margin of Tolerance (MOT).

The 2014 scheme builds on previous measures in this regard. A company may be permitted to retain up to 50% additional profit before making additional payments to the government for excessive profits. In turn, no company will be permitted any price increase to address lack of profitability unless its profits have fallen below 50% of allowable profit (ABPI Report 2014).

Greece applies price control for all medicines as Turkey. The price of imported and domestic medical products mustn’t exceed the minimum ex-factory prices in Europe. France applies direct price control for medicines sold in hospitals, while reimbursement prices are determined by manufacturers and price controls are implemented for reimbursed medicines (Balçik and Karsavuran 2012).

It is highly difficult to keep the reimbursement prices ‘lifetime’ under current circumstances. Prices set for the launch of the drug is maintained for a certain time and then the price is defined according to the price criteria again. Most countries prefer price cuts instead of increasing the price of medicines. Direct price controls may lead to a retrenchment in drug prices by slowing down the increase in drug prices or by reducing the price of some drugs. However, the prices of medicines in the mentioned countries usually continued to increase. This increase is considered to be caused by the increase in the amount of medicine consumption or new molecules added to reimbursement lists (Mossialos et al 2004).

## Conclusions

It is impossible to avoid the changes in the health care field all over the world. Technological developments, changes in the epidemiological patterns, increasement of the world population, rapid growth of society’s expectations and innovations in treatment processes cause health to be a area that is much more dynamic than the others. Keeping up with this major evolution has, accordingly, come to be the primary need of each country. Health area has strategic importance. By the virtue of this area, will a community be one that is healthy, more productive, self-sufficient and sustainable.

It is also of great importance in our country for short, medium and long-term measures to be implemented for the sake of developing R & D and entrepreneurship ecosystem. R & D economic zones with special infrastructure, like in the example of other countries in the world, should be established in order help Turkey achieve her aim to develop new molecules and to produce drugs with a higher added value (TITCK 2014).

It can be seen that the R & D activities take an important place in enhancing the market access of innovative medicines. It is also obvious that, for R & D activities, there is a need for strong public-pharmaceutical industry-university collaboration. Regulations in this respect have undergone frequent changes in recent years but the impact of these changes should be examined in the long term. Further research into the relation between the box and value-based changes occurring in drug consumption and the changes in the regulations will also contribute to the arrangements to be made in the future.

A fixed rate of 1.9595 for Euro is applied between 2009 till 2015 on reference pricing in Turkey. This rate was changed to 2,00 on June 2015 and with the latest price decision (2015); the value of 1 (one) Euro in Turkish Liras to be used in the pricing of medicinal products for human use shall be designated upon multiplying with the adaptation coefficient designated as 70 percent of the average annual Euro value to be calculated upon taking into basis the daily indicative Euro foreign exchange sales rate realizations of the Central Bank of the Republic of Turkey declared on the Official Gazette of the previous year. (Decision on The Pricing Of Medicinal Products For Human Use 2015)

It is an undeniable fact that the long-standing policy of Turkish government to supply cheap drugs has many benefits for the budget in the short term. This can be seen clearly in the short term outcomes of the drug budget implementations.

Turkey is an important market for pharmaceutical sector with a population of over 75 million. Government policies that would be implemented in pricing and reimbursement processes may result positive effects to access of medicines in the short term and in the long term, but in order to prevent drug shortages and access to therapies Turkish Government has been taking important decision such as localization of medicines and increasing euro value from 1,9595 to 2,1186 TL.

It is normal to observe a growth in health sector in a country that is growing in economic terms. However, considering the medicine particularly, the limits set by global budget did not seem to correlate with the economic growth of Turkey. The global budget implementation has stopped end of 2014.

‘‘Price’’ stands out as an important element in the plans of pharmaceutical industry to realize new projects and investments to improve the competitiveness of the market and make new investments. Pricing is expected to be based on an equitable, transparent and lucid system. Drug policies are expected to be in same way with the national policy of the country and the parameters of clarity and efficiency, equity and cost should have an impact on all aspects of evaluations from the beginning.

Strict price controls appears to be effective on the point of costs. However, the cheapest price policy as the only policy may have negative consequences in other circumstances, and just because the price planning happen to be successful does not mean a similar success would be reached in total health expenditure as planned. It is a well-known fact that rational drug use comes before drug prices. So, supporting rational drug use is really important. Focusing on demand side for efficiency, equity and quality improvement would give us rational drug use. Innovative drugs will increase drug expenditures in the future (Balçik and Karsavuran 2012).

Determining drug prices is not an easy decision after all and as it is directly affected by the internal dynamics of the country, no country can be expected to have the same conditions as any other country in this respect. However, as an emerging market, one of the priorities of Turkey aimed to be becoming the pioneer country of pharmaceutical market as both a producer and an adminiştrator. This would definitely contribute to the general economy of the country in the long term. It is recommended that the figures which may be a burden on the economy in the short term be compensated in the long term with the investment agreements that will be signed with the industry. At this point, an example action that may be recommended is taking the average of the three lowest drug prices as reference, like in Greece; rather than picking the lowest price.

Greater cross-disciplinary interaction among economists, ethicists, and physicians can help to reduce the disjunction between innovation and access and can improve access and patient care. Increased interaction between experts in this multi-disciplinary area would result in better access to innovative medicines and treatments for patient care together with better management of drug price settings (Lue et al. 2015).

Creating the inter-ministerial commission and making the pricing and reimbursement decisions through that commission, as seen in the other countries in our study, would minize problems between the institutions and lead to a healthier decision making process. It is thought that employing full-time experts will increase the working capacity and productivitiy of the commission. The primary objective here should be keeping the technical and scientific capacity of the Commission at the highest level, as in other countries. Then it would be feasible to draw clear lines between the health service provision, payer and decision-makers.

## Key messages

1. Healthcare expenditures rises in all over world because of expensive therapies

2. Each country has specific regulations and pricing and reimbursement policies of medicines based on economic situation reimbursement methods and market size

3. Reimbursement considerations in EU countries based on economical evaluations and budget impact

4. Pricing and reimbursement policies of medicines should be aimed to show compliance with efficient and sustainable health policies.
